# Nurses’ and pharmacists’ learning experiences from participating in interprofessional medication reviews for elderly in primary health care - a qualitative study

**DOI:** 10.1186/s12875-017-0598-0

**Published:** 2017-02-28

**Authors:** H. T. Bell, A. G. Granas, I. Enmarker, R. Omli, A. Steinsbekk

**Affiliations:** 1 Department of Pharmacy, Faculty of Health Sciences, Nord University, Namsos, Norway; 2 Department of Public Health and General Practice, Norwegian University of Science and Technology, Trondheim, Norway; 3 School of Pharmacy, University of Oslo, Oslo, Norway; 4 Centre for Care research Mid- Norway, Steinkjer, Norway; 5 Department of Nursing, Mid University, Østersund, Sweden

**Keywords:** Medication review, Nurse, Pharmacist, Learning, Inappropriate drug use, Primary care

## Abstract

**Background:**

Traditionally, drug prescription and follow up have been the sole responsibility of physicians. However, interprofessional medication reviews (IMRs) have been developed to prevent drug discrepancies and patient harm especially for elderly patients with polypharmacy and multimorbidity. What participating nurses and pharmacists learn from each other during IMR is poorly studied. The aim of this study was to investigate nurses’ and pharmacists’ perceived learning experience after participating in IMRs in primary health care for up to two years.

**Methods:**

A qualitative study with semi-structured focus group interviews and telephone interviews with nurses and pharmacists with experience from IMRs in nursing homes and home based services. The data was analysed thematically by using systematic text condensation.

**Results:**

Thirteen nurses and four pharmacists were interviewed. They described some challenges concerning how to ensure participation of all three professions and how to get thorough information about the patient. As expected, both professions talked of an increased awareness with time of the benefit of working as a team and the perception of contributing to better and more individual care. The nurses’ perception of the pharmacist changed from being a controller of drug management routines towards being a source of pharmacotherapy knowledge and a discussant partner of appropriate drug therapy in the elderly. The pharmacists became more aware of the nurses’ crucial role of providing clinical information about the patient to enable individual advice. Increasingly the nurses learned to link the patient’s symptoms of effect and side effect to the drugs prescribed.

**Conclusions:**

Although experiencing challenges in conducting IMRs, the nurses and pharmacists had learning experiences they said improved both their own practice and the quality of drug management. There are some challenges concerning how to ensure participation of all three professions and how to get thorough information about the patient.

**Electronic supplementary material:**

The online version of this article (doi:10.1186/s12875-017-0598-0) contains supplementary material, which is available to authorized users.

## Background

Elderly living at home and in nursing homes use many drugs [1] and are therefore at risk of experiencing adverse drug reactions and increased risk of falls [2]. Physicians have traditionally been responsible for drug prescription and follow up, but it has e.g. been shown that they renew prescriptions without assessing if the medication is still indicated [3]. In addition frequent changes in caregivers both between secondary and primary care but also within primary care, make elderly patients and patients with complex care needs more vulnerable to drug discrepancies that can lead to drug errors [4]. As a result systems for medication reconciliation and interprofessional medication reviews (IMRs) have been developed [5]. IMRs by physicians, nurses and pharmacists have been showed to reduce drug-related problems and improve quality of prescribing in hospitals and nursing homes patients [6, 7].

Primary health care workers often face additional challenges compared to those working in a hospital setting due to lack of geographical proximity of the team members [8]. Facilitators and barriers to interprofessional collaboration in primary health care has been identified as being both structural and cultural like the need of shared facilities, written procedures, shared communication tools, accessibility, trust, value and leadership [9, 10]. Collaboration between nurses and community pharmacists in primary care concerns mainly product advice and dispensing issues [11], but when nurses and pharmacists collaborate in an inpatient medical setting they can learn to appreciate each other’s roles [12].

The existing research on IMR has mainly focused on the outcome of the intervention of the drug-related problems [13] or the different participants’ perception of the collaboration process [14]. However, we have found no research focusing on what nurses and pharmacists perceive to learn when participating in IMRs. The aim of this study is therefore to describe what nurses and pharmacists perceive to learn from participating in interprofessional drug review teams in a primary health care setting for up to two years.

## Methods

This qualitative study is part of a larger study with focus group and individual interviews performed between October 2014 and February 2016 in Norway. The Regional Committees for Medical and Health Research Ethics in Central Norway approved the study (2014/1140).

### Setting, training and practice

In Norway the municipalities are responsible for social welfare and health care for all its inhabitants, including home based health and social care and nursing homes [15]. Part-time contracted general practitioners (GPs) most commonly provide the medical services in nursing homes [16] and the home dwelling elderly with home based health and social care receive their medical service from their GP with assistance from home care nurses [17]. The nurses in home care services often work alone as nurses, supported by staff with less or no formal nursing education [18]. The majority of Norwegian pharmacists work in privately owned community pharmacies or hospital pharmacies. The municipalities have contracts with a hospital or community pharmacy to provide services to inspect drug management or to perform medication reviews [19].

Interprofessional medication reviews is not established in primary health care in Norway, but since 2013 the GP legislation states that patients prescribed four or more drugs, the GP should perform medication reviews if this is necessary from a medical point of view [20]. There is yet no such legislation for patients in nursing homes. In 2011–13 the Norwegian Patient Safety Programme “In safe hands” was implemented throughout Norway. Two of the 12 focus areas were to establish interprofessional teams on medication reviews in nursing homes and home based health and social care services [21]. The centres for Development of Institutional and Home Care Services [22] in each of Norway’s 19 counties were responsible for spreading the program to municipalities in their own county, following a national guideline based on the Intergrated Medicines Management (IMM-model) [23].

The IMM-model consists of four main steps [23] and is based upon the original version from Northern Ireland [24]. In the first step, the nurses interview and go through a checklist with the patient, order blood samples and construct a drug list based on the available information. In the second step, the nurses pass this information to the pharmacist who identifies potential drug-related problems and checks if the prescribing is according to national guidelines. In the third step, the drug review is performed at a case conference where the responsible physician, nurse and the pharmacist meet and perform medication reconciliations and reviews where they discuss the best drug regime for that specific patient. The physician is responsible for the overall treatment. Finally, the nurse updates information of the drug regime agreed upon in the patient’s journal. They also observe how the patient responds to any changes and give feed back to the GP when necessary [21]. The drug reviews require consent from the patient that allows health information to be shared in between the three professions involved.

The municipalities were encouraged to form interprofessional teams, consisting of at least one representative from the three professions; physician, nurse and pharmacist. In a course consisting of three structured learning meetings throughout one year the interprofessional teams of health professionals, were introduced to the methodology in the IMM-model, introduction to why IMRs are useful for the elderly patient, encouraged to initiate interprofessional cooperation and to establish interprofessional medication reviews (IMRs) [25]. The interprofessional teams were encouraged to start practicing medication reconciliations and IMRs after the first meeting in the course [21]. The nurses within each team were charged with developing local routines for the selection of eligible patients, routines for how to organize IMR-tasks on top of everyday tasks, and how to book case conferences. They were also responsible for spreading of knowledge on IMR to their colleagues. Only two physicians from the 11 participating municipalities attended the implementation course and only at the first meeting. It was therefore up to the team leaders, who were nurses, to recruit an appropriate physician from their municipality to their team. In some of the municipalities no physician was recruited and the IMRs were performed with only nurse and pharmacist present. In these teams the pharmacist first presented her findings to the nurse who then gave her input before she later was responsible of presenting the revised results from the discussion to the physician.

### Informants and data collection

We aimed to recruit physicians, nurses and pharmacists who had participated in the patient safety program and who had experience of performing IMR. To ensure a representative sample, we wanted to have teams representing different municipality size, different length of experience with IMR and from both nursing homes and home based health and social care. The reports given by the different teams after the course were used to select teams based on these criteria.

To recruit informants, the appointed team leaders in 11 municipalities in Central Norway were contacted by e-mail and then by phone. They were told that they could volunteer teams even though not all team members in each team wanted to participate. This approach only lead to the recruitment of two pharmacists participating in several teams each and therefore additional two pharmacists were recruited through the hospital pharmacies in the county.

The semi-structured focus group interviews were conducted with representatives from all included teams 1–2 years after initiation of the course in their county. Focus group is particularly useful for exploring people’s common experiences, attitudes and views in environments where people interact. The use of group interaction is an explicit part of the method [26]. The focus group interviews were either conducted at a nursing home or at the city hall in the municipality. An interview guide with open-ended questions focused on the following themes was used; perceived learning and gained knowledge in addition to perceived facilitators and barriers to be able to perform interprofessional medication reviews in primary health care [27] (Additional file 1). The focus group interviews lasted approximately one and a half hour, were digitally recorded and led by the first author (HTB). The telephone interviews lasted approximately 20 min performed by the first author using the same interview guide. Participants were provided with written and oral information about the study and informed that they could withdraw at any time. Written informed consent was obtained from the participants before the interviews were conducted.

### Data analysis

The interviews were digitally recorded and transcribed verbatim. They were analysed using the method of systematic text condensation [28], according to an iterative four-step process. In the first step, all authors read a selection of the transcripts to identify preliminary themes, which were discussed. In the second step, the transcripts were searched in detail by the first author to identify meaning units, which were sorted under the preliminary themes and these were presented to the other authors. In the third step, the meaning units were arranged into subthemes. In all these steps the preliminary themes were adjusted. Then a narrative condensate was made of the meaning units sorted under each theme and subtheme. In the last step, an analytic text was produced based upon each theme and subtheme. The themes and the analysis were discussed among the authors several times and also in an extended research group to ensure validity. During the whole process, the authors went back to the original transcripts to ensure that the analysis was based upon them.

## Results

A total of thirteen nurses from five different nursing homes and three home-based care units and four pharmacists were interviewed. There were three focus group interviews consisting of nurses only but from both nursing homes and home based care, and two with nurses from different workplaces and a pharmacist. The remaining two pharmacists were interviewed by telephone. Further participant characteristics are presented in Table 1

**Table 1:** Participant characteristics

	Nurses (*n* = 13)	Pharmacists (*n* = 4)
Working in nursing homes	8	-
Rural	5	3
Urban	3	4
Working in home-based care	5	-
Rural	2	-
Urban	3	-
≤1 year experience of performing IMR in primary health care	5	1
>1 year experience of performing IMR in primary health care	8	3
Experience from performing IMR in hospital	-	3
Experience of performing IMR with a physician present	10	4
Experience of performing IMR without a physician present	3	3

.

The perceived learning from participating in structural interprofessional medication reviews in primary health care are arranged in the following five themes; Learning about each other’s role, A more comprehensive documentation of drug management, Challenge the physician’s role, Importance of detailed information about each patient and Linking patient’s symptoms and medication use.

### Learning about each other’s role

It was new for the nurses in the nursing home and home based health and social care to learn during the interprofessional medication reviews (IMRs) that pharmacists could provide advice and guidance on appropriate drug use for the elderly patients. This was contrary to their previous experience of pharmacists as someone who came on irregular visits and primarily focused on controlling their drug management routines. After taking part in IMRs, however, they now perceived the pharmacist as a supportive partner who could give them useful advice on pharmacology and pharmacotherapy. They especially appreciated the pharmacists’ knowledge concerning drug monitoring data for laboratory values like haematology, proteins, hormones, vitamins and drugs such as digoxin with a small therapeutic window. The pharmacists said that after the establishment of the IMR-teams, the number of telephone and e-mail inquires from both nurses and physicians regarding drug therapy questions had increased.

*The pharmacists gave us a very good impression by showing how much they could contribute regarding knowledge on drugs and drug therapy. They knew much more than we thought they did. Our previous impression was that they sold plasters and handled the drugs at the pharmacies. (Nurse, less than one year of experience with IMR)*

The pharmacists did not meet with the patients themselves and therefore talked about a dependency on the patient information given by the nurses. Preparing for the IMRs could be challenging for the pharmacists when not having access to updated drug monitoring data and complementary patient documentation. They perceived the majority of the nurses to provide good information and documentation, but there were also examples of the contrary like e.g. nurses who did not know the patient well.

*“A case has many sides and I only know the patient through his drug list. So it is very important for me to get the additional information from the nurses. Like when a patient has pain. When does he have pain and what type of pain?” (Pharmacist, more than 2 years experience of IMR)*

### A more comprehensive documentation of drug management

Taking part in the IMR, the nurses talked about how they learned to become more critical towards their own drug management routines and talked about a raised awareness on better documentation of these routines in everyday work. In addition, especially the nurses working in home based health and social care, learned the importance of medication reconciliation that ensured an updated list of drugs in use due to the high number of carer that could be involved. An updated drug list which they trusted to be correct also helped them to get a more complete and documented overview of the patient’s medical situation and to later link this to the drugs in use.

When the other professions regarding drug management raised challenging questions the nurses said they learned the need for accurate, updated and detailed information in the patient journals about drugs in use and the need for a broader focus on drug management as a whole. This included having all the patient’s diagnosis listed in the journal and to ensure written indications for the different drugs to be available for all health personnel involved with the patients. Participating in IMRs were therefore said to promote an understanding of comprehensive documentation of the drug management as a nurse task just as important as the other nursing tasks. It was highlighted that staff without any formal nursing education, who often are the ones to hand out the drugs and spend most time with the patients, especially appreciated this quality improvement.

*“In the beginning when the indications were vague and not always written on the patient’s medicine card it was difficult to evaluate the usefulness of the drugs. Especially since it was not written why they were put on those drugs.” (Nurse, less than one year of experience of IMR)*

### Challenge the physician’s role

The nurses with experience of performing IMRs together with both a physician and a pharmacist said that the pharmacist challenged the physician’s role as the only drug expert. In particular this involved posing other types of questions, comments and solutions than the nurses did. This was said to stimulate the physicians to reflect upon their previous drug prescribing and in some instances forced the physicians to argue their case when there where disagreements. Both professions perceived disagreements as strength for the quality of drug therapy for the patients, because it triggered the physician to review the drug therapy choices initiated by themselves or other prescribers. Some nurses felt that the pharmacists’ questions echoed comments and questions previously raised by themselves to the physicians, but where they hitherto had failed to argue their case or gave in without getting a clear answer. However, when the pharmacists asked questions during the team discussions the physicians responded better and more clearly.

*“The pharmacist sees it from another angel and uses her own specialist knowledge to come up with new alternatives that the physician has not thought of – as far as I can see that must increase the quality.” (Nurse, with more than one year experience of IMR)*

The nurses that had performed IMRs without having a physician present did not compliment the pharmacist in the same way and said that the physician was the one who knew what was best for the patient regardless the pharmacist’s suggestions. These nurses sometimes perceived the physicians as headstrong but it was also emphasized that the physicians often had long experience in the municipality and therefore had a better insight into the totality of the patient’s situation. In some of the cases the experience was also that when the nurses presented the suggestions to the physicians after the IMR with the pharmacist the physician rarely if at all took the suggestions into account.

*“We presented it to the physician. And since they were only suggestions he did not go for them.” (Nurse, with less than one year of experience of IMR and IMR without physician)*

The pharmacists that had experienced IMR without a physician appreciated the nurse’s contribution during the drug review, but found it unsatisfactory not being able to discuss and argue their case directly with the physician. Not knowing whether their suggestions were followed were also highlighted as a disadvantage since they perceived to learn less when missing out the discussions with the physician in particular. When the physician was present the pharmacists perceived that the physicians in the majority of the cases appreciated their contributions, but there were also experience of the contrary. With time the pharmacists said to understand better why their theoretical grounded suggestions not always were accepted by the physicians, mainly due the physicians’ knowledge of a larger totality of the patients’ situation than themselves. This was said to contribute to a wider understanding of some of the choices taken by the primary care physician and to enable the pharmacists to view a case from another perspective than they usually did.

*“We get the physicians view of the patient. A GP know the patient and his history better than I do and I might suggest a change that might have been tried out before (…) which the physician find difficult to implement (…) because the patient might refuse.” (Pharmacist, more than 2 years experience of IMR)*

### Importance of detailed information about each patient

In some municipalities the pharmacists experienced that the nurses struggled to find time to do their part of the preparatory work, such as interviewing the patient, order drug monitoring blood samples and filling in the patient checklist. This resulted in delayed or deficient documentation to the pharmacist. These drug reviews were perceived as unsatisfactory since the pharmacist then only could give generic advice and not tailor the suggestions for the patient in question.

*“The advices we give might be good, but it might not be the best for that specific patient. For example I set up an optimal list of drugs based upon the guidelines, but then maybe the patient is not able to swallow tablets or remember to take the tablets twice a day. There is a lot of extra information I need to be able to set up an appropriate list of drugs.” (Pharmacist, more than 2 years experience of IMR)*

As a consequence, one pharmacist had changed the preparing routines prior the IMR and now spent the whole day at the nursing home or home based health care. Information about the patient were gathered and collected by the pharmacist using information from the patient’s journals and talking to the nurses. The preparations took place in the morning and then the IMR was performed in the afternoon. This was said to give a better access to the existing documentation and also gave the pharmacists the opportunity to ask the nurses and other staff of supplementary information when needed.

None of the drug reviews were performed with the patient present. Perceiving themselves as the patient’s voice at the drug review made the nurses discover that detailed knowledge of each patient was a necessity to be able to answer questions raised by the other two professions at the IMR. Contrary, the nurses felt awkward when presenting patients they did not know well or relied on second hand information. Good cooperation with nursing assistants or other care workers was perceived as important when collecting necessary information on function level and behaviour. Likewise, it was said to be important to discuss the observations of each patient in the nurse collegium since different persons perceived the patients differently. This was especially important in home based health care service as opposed to nursing homes, because the nurses spend only a short time with domiciliary patients.

It was also expressed as difficult to convey patient information, when the nurse interviewing and collecting the information about the patient might not be the same presenting at the drug review. The teams that perceived good backing for the IMR tasks in the municipality and who had managed to develop good routines throughout the collegium were also those who found collecting these data least difficult.

*“I felt sometimes – oh I should have known more about this patient. I do not believe that I will be the one that continues performing drug reviews.” (Nurse, less than one year experience of IMR)*

### Linking patient’s symptoms and medication use

The pharmacists experienced that the nurses gradually showed a deeper engagement for the medication reviews, such as being more updated on the patients’ conditions, symptoms and the prescribed drugs. The nurses said that during the medication reviews they had learned new things about pharmacotherapy, especially how drugs work and drug-drug interactions. Examples were knowledge about drugs with anticholinergic effect and drugs that can increase the risk of falls in their patients.

*“We have learned more about combination of different drugs and anticholinergic effects. (…) Being more aware on pain relief – the need to assess the treatment more often and at an earlier stage. Previously they had Paracetamol 1 g x 3 without us assessing, but now we ask them whether they still need them. The questions pop up more frequently.” (Nurse, more than one year experience of IMR)*

A stronger knowledge on pharmacotherapy made the nurses more observant and capable of interpreting patients’ behaviour possibly linked to the drug use – both effects and side effects. They said that they became more curious and critical, therefore asking more questions to the physicians and pharmacists. They also became more aware of the need for a more comprehensive documentation of the drug management. New awareness was said to be transferrable to other patients not yet part of IMRs such as assessing drug therapy at an earlier stage, for example in long-term pain treatment. Participation in IMR with both pharmacist and physician heighten their awareness on drug treatment as a whole and were said to contribute to the perception of more individual care.

*“We have gained a greater awareness on drugs (…) You become a little more aware when you see a drug sheet. “Can this be correct?” (…) You become more critical.” (Nurse, with more than one year experience of IMR)*

When asked if the learning emerged from participating in the course or from performing IMR, both the professions linked the learning to active participation in IMRs. They said that at a course you were only a passive recipient, whereas during IMR you had to use your adopted knowledge actively which again led to learning. Arguing their case was particularly highlighted to contribute to learning. The nurses who had performed IMRs without a physician present spoke less of what they had learned during this period.

*“I believe that IMR give something extra since you have to use what you know actively. You get forced to think through what you are doing. Why do we do this IMR, and you look at the check list and think of the patient’s drugs and how the whole situation is for the patient.” (Nurse, more than one year experience of IMR)*

## Discussion

In this study it was found that both professions reported to learn more about each other’s role when performing interprofessional medication reviews (IMRs). The nurses’ perception of the pharmacist changed from being a controller of drug management routines towards being a source of pharmacotherapy knowledge and a discussant partner for appropriate drug therapy in the elderly. The pharmacists became more aware of the nurses’ crucial role of providing clinical information about the patient to enable individual advice. Increasingly the nurses learned to link the patient’s symptoms to the prescribed drugs due to having learned more about pharmacology and pharmacotherapy and also the importance of comprehensive drug management and detailed information about each patient. With time both professions jointly spoke of an increased awareness of the benefit of working as a team and the perception of contributing to better and more individual care. Through this they learned to challenge the physicians’ knowledge and prescribing decisions. IMRs were found to be unsatisfactory without the physician’s input and without thorough information about the patient’s condition.

### Learning from each other and the experience of mutual interdependence

Others have found that pharmacists can have other roles than controlling and checking up on the other professions’ drug handling [29] and that other professions’ awareness of the pharmacists’ clinical skills increases with time [12, 30]. This is in line with our findings. The most prominent learning reported by the informants in this study was how they came to appreciate each other’s role during the medication reviews and how this created a sense of mutual interdependence. Participating in IMR were said to lift the focus on medication management as an important nurse task and that the pharmacists’ contributions during the IMRs elevated the nurses’ own performance. Nurses and physicians have both stated a perceived elevation of performance and educational benefit from working together with pharmacists [12].

It has been found that effective teamwork demands role clarity and an understanding of roles and responsibilities [8, 31], where working together can create a sense of mutual interdependence when different professions learn to know each other’s roles [32]. Pharmacists cooperating with other professions have been shown to facilitate a team approach that improved the patient’s drug related outcomes [12, 30]. This is in line with our study. The discussions during the IMRs where all professions were present were especially perceived as beneficial and therefor indicate that doing IMRs together can contribute to both learning and the perception of mutual interdependence.

### Challenges when applying IMRs in primary health care

Lack of mandate for the pharmacist’s role [10], the time the pharmacist was on site and funding of the pharmacists [14] has been found as barriers for pharmacists participating in interprofessional teams in primary health care. The model of IMM provides guidelines for the role of the pharmacists in IMRs [23], but the funding is dependent on the municipalities’ willingness to pay for the pharmacist and can be a limitation for the continuation of IMRs in primary care. Our findings also concur with findings in studies from Supper et al. and Bell et al. which found that limited access to the complete medical history and relevant monitoring data can be perceived as a barrier for the pharmacist [10, 33]. In our study it was particularly evident that the main barrier was if there were delayed or deficient documentation about the patient’s condition given to the pharmacist prior to the IMRs.

The physician has a pivotal role in decisions making about the prescribed medicines [34]. No surprise, when the physician is not present at the IMR, the interviewees said that they learned less. Accessibility has been shown to be a premise for interprofessional collaboration particularly between physicians and allied health professionals [9]. Not having all professions present is also a deviation from the IMM-model [24]. When team members have separate bases or buildings they are less integrated with the team [8]. However, it was perceived as challenging to gather all professions for joint meetings in primary care. The same was found in studies of interprofessional cooperation in family health teams and family medicine clinics that describe challenges according to management, leadership, time, space and governance [32, 35]. Thus, there seems to be a need for innovative solutions to overcome obstacles such as finding common time and booking meeting facilities for the case conferences, in home based care and in rural municipalities.

Another challenge experienced by our informants was how to ensure good and correct information about each patient. Shift work and part-time positions in addition to nurses spending little time with home dwelling patients, made it difficult to collect the relevant patient information. Thus it can be a challenge to gather and collect comprehensive and objective information about the patient from all personnel involved prior to the medication reviews. This raises the question whether the patients themselves, unlike today [34], should be present during IMR to make sure that the patients’ perspectives are taken into account. From an ethically perspective patients should be included in decisions about their own care [36]. We have, however, not found any studies investigating such a solution in IMRs.

### Medication management in primary care – more than right medicine to right patient

Since service users in primary care receive lower level of medical service intensity compared to hospital patients, the need to observe, document and report effects of the medical treatment has been reported to be an even more crucial task for the nurses [37, 38]. This includes monitoring medication administration, adherence and the effect medicines have on patients’ symptoms [39]. The findings in this study indicates that this could be problematic due to the infrequent contact with the patient in home based care and not being challenged to report specifically on these issues. It is therefore reassuring that the IMR was experienced as an arena where the nurses became more aware of the importance of thorough medication handling routines and a need for written high quality instructions on all the steps in the medication management process.

### Strengths and limitations

The strength of this study was that the informants had real life experience with doing interprofessional medication reviews over time and the variation in the clinical situations the IMRs were conducted. In addition, there were variation in geography and population. It was a limitation that the interviewees came from one county in Norway and none of the municipalities were a large city. However, as others have similar findings [12, 30] this does not seem to limit the transferability. The lack of the physicians as informants is another limitation. The physicians were invited on equal terms as the other two professions, but none of the physicians involved responded to the invitation. There is therefore a need for studies in the physicians’ perspectives but also on the patients’ perspective.

The focus groups purposefully consisted of teams that had participated in the course and performed medication reviews together. This contributed to a relaxed and freely speaking environment. Former disagreements could have limited discussions of topics they knew could cause disagreement and even hamper future collaboration. Furthermore, the fact that HTB is a pharmacist could have limited criticism of the pharmacists’ role. However, the review of the transcripts indicates that the interviewees spoke also about disagreements during the interviews.

From the interviews, it seemed like the nurses learned more from the pharmacists than the other way around. This is likely to be due to the predominance of nurses among the informants, but it might also be due to the nurses getting access to a new profession’s knowledge and skills, which was unlike the pharmacists whom the majority had former experience from IMR in hospitals.

## Conclusion

From the nurses’ and pharmacists’ perspective in this study, IMRs in primary health care can be a learning arena for the participating professions. It was experienced to contribute to improving their own practice and the quality of drug management, resulting in better and more individualised care. There are some challenges especially concerning how to ensure participation of all three professions and how to get thorough information about the patient.

## Additional file

Additional file 1: 20170203InterviewguidemanuscriptIMREndelig.docx, Interview guide. (DOCX 15 kb)

## Abbreviations

GP: General practitioner
IMM: Integrated medicines management
IMR: Interprofessional medication review
