# Supplementary material for: Nurses’ and pharmacists’ learning experiences from participating in interprofessional medication reviews for elderly in primary health care - a qualitative study
Source: BMC Fam Pract. 2017 Feb 28;18:30. doi: 10.1186/s12875-017-0598-0 (PMC5330158; doi:10.1186/s12875-017-0598-0)
Supplement: Additional file 1: — 20170203InterviewguidemanuscriptIMREndelig.docx, Interview guide. (DOCX 15 kb) [file 12875_2017_598_MOESM1_ESM.docx]

Interview guide:

1. From your experience of performing Interprofessional Medication Reviews (IMRs) in nursing homes and home-based care, can you describe factors that promoted or hindered the process of working together?
2. Can you describe the knowledge and the learning you gained by performing IMR in primary care?
   1. What were your expectations towards the other professions, i.e. physicians, pharmacists and nurses, and their contributions into the process of IMR?
   2. How did you experience the communication, information flow and the collaboration between the different professions?
   3. What was in your view the unique contribution from the different professions during the IMR?
   4. Did you beforehand have any expectations of what the results from an IMR would be to patients and professionals?
